# Validation of an arterial tortuosity measure with application to hypertension collection of clinical hypertensive patients

**DOI:** 10.1186/1471-2105-12-S10-S15

**Published:** 2011-10-18

**Authors:** Karl T Diedrich, John A Roberts, Richard H Schmidt, Chang-Ki Kang, Zang-Hee Cho, Dennis L Parker

**Affiliations:** 1 Utah Center for Advanced Imaging Research, Department of Radiology, University of Utah, 729 Arapeen Drive, Salt Lake City, UT 84108, USA; 2 Department of Biomedical Informatics, University of Utah, 26 South 2000 East Room 5775 HSEB, Salt Lake City, UT 84112, USA; 3 Department of Neurosurgery, University of Utah, Health Science Center, Bldg 550, 5th Floor, 175 N. Medical Drive East, Salt Lake City, UT 84132, USA; 4 Neuroscience Research Institute, Gachon University of Medicine and Science 1198, Kuwol-dong, Namdong-gu, Incheon, 405-760, Korea

## Abstract

**Background:**

Hypertension may increase tortuosity or twistedness of arteries. We applied a centerline extraction algorithm and tortuosity metric to magnetic resonance angiography (MRA) brain images to quantitatively measure the tortuosity of arterial vessel centerlines. The most commonly used arterial tortuosity measure is the distance factor metric (DFM). This study tested a DFM based measurement’s ability to detect increases in arterial tortuosity of hypertensives using existing images. Existing images presented challenges such as different resolutions which may affect the tortuosity measurement, different depths of the area imaged, and different artifacts of imaging that require filtering.

**Methods:**

The stability and accuracy of alternative centerline algorithms was validated in numerically generated models and test brain MRA data. Existing images were gathered from previous studies and clinical medical systems by manually reading electronic medical records to identify hypertensives and negatives. Images of different resolutions were interpolated to similar resolutions. Arterial tortuosity in MRA images was measured from a DFM curve and tested on numerically generated models as well as MRA images from two hypertensive and three negative control populations. Comparisons were made between different resolutions, different filters, hypertensives versus negatives, and different negative controls.

**Results:**

In tests using numerical models of a simple helix, the measured tortuosity increased as expected with more tightly coiled helices. Interpolation reduced resolution-dependent differences in measured tortuosity. The Korean hypertensive population had significantly higher arterial tortuosity than its corresponding negative control population across multiple arteries. In addition one negative control population of different ethnicity had significantly less arterial tortuosity than the other two.

**Conclusions:**

Tortuosity can be compared between images of different resolutions by interpolating from lower to higher resolutions. Use of a universal negative control was not possible in this study. The method described here detected elevated arterial tortuosity in a hypertensive population compared to the negative control population and can be used to study this relation in other populations.

## Background

There is evidence that hypertension can affect blood vessel morphology. Increasing stage of hypertension has been shown to correlate with increased tortuosity or twistedness of white matter arterioles in autopsy photomicrographs [1]. In vitro studies on extracted dog arteries showed increasing blood pressure caused increases in tortuosity [2]. In contrast, one study in Korea found that while the number and branches of lenticulostriate arteries visible in Magnetic Resonance Angiography (MRA) images decreased in hypertensive subjects compared to negative controls, an increase in tortuosity was not seen in tortuosity projection measurements made on 2D projections of the 3D data [3].

Tortuosity measurement has the potential to quantify morphological changes in arteries due to hypertension. Tortuosity can be measured from MRA images of arteries. The process starts with MRA imaging of arteries, segmentation of arteries, calculation of centerlines, and calculation of tortuosity from the centerlines. Centerlines simplify arteries and other tubular structures to a single line passing through the middle of the artery making measurement of length and position possible. Measurements on the centerlines can be used to calculate tortuosity scores.

The most commonly used tortuosity measure is the distance factor metric (DFM) that requires two end-points to measure the ratio of the length *L* along the centerline and the distance *d* between two end-points [4-10]. The DFM suffers some weaknesses. Some arteries only have one anatomical end-point in an image volume and local tortuosity scores can rise and fall along an artery. The DFM can miss local tortuosity depending on the selection of the two end-points. Furthermore, the comparison of DFM tortuosity between multiple subjects can be challenging when the image volumes do not all share the same two anatomical centerline end-points.

Centerlines can be calculated by Dijkstra's algorithm [11] which finds the shortest or lowest cost path from any given point in the arterial segmentation to a selected goal point or node. Each voxel (three dimensional pixel) of the arterial segmentation is assigned a cost based on its position with respect to the goal. The longest lowest cost paths from the distal ends of the arteries back to a central goal node are the centerlines [12]. The selection of the central goal node and cost function can affect the path of the centerline.

Existing images from previous studies and clinical scans provide a large set of data for analysis that saves the time and cost of acquiring new images. Reusing existing images for comparison studies may present difficulties if the images have been acquired with different parameters including the field strength of the magnetic resonance imaging (MRI) scanner, resolution, and field of view (FOV) placement. FOV placement may affect whether the same artery segments are seen in both views. Differences in resolution may affect the tortuosity measure. Some vessels visible in MRA images from high 7.0 T field strengths may not be seen at lower resolution and the high field may cause phase flow artifacts [13,14] that can be mistaken for arteries by centerline algorithms, requiring pre- or post-processing for removal. Filtering can cause data loss and could affect the tortuosity measure. Negative controls may be obtained from existing images from patients with non-vascular diseases but proof of being truly negative is needed.

Testing of centerline and tortuosity measurement algorithms can be conducted on numeric phantoms. Numeric phantoms are three dimensional shapes generated by equations in computer software with known morphology and centerlines. Algorithm calculated centerlines can be compared to known centerlines to assess accuracy and tortuosity measures can be tested on different shapes with known tortuosity.

In this study we first test the stability and accuracy of our Dijkstra’s shortest path centerline algorithms by using different cost functions and goal node voxels on numeric phantoms and a sample of brain MRA images. We modified the classical DFM tortuosity measurement to create a tortuosity curve that provides additional information and tested the measurement on numeric phantoms. We applied the DFM tortuosity curve measurement to existing brain MRA images. The images included data on the same subjects filtered for noise with different filters and at different resolutions to test the effects of filtering and resolution on tortuosity. Hypertension data from the Korean hypertension study [3] and clinical hypertension data from Utah were tested to determine if the method can detect a correlation between hypertension and tortuosity of the arteries visible in MRA images. Tortuosity was also compared between three negative controls to test similarity and determine if universal negative controls can be used.

## Methods

### Image analysis flow

The image data is analyzed by interpolation, filtering, segmentation, centerline extraction, tortuosity curve calculation, and reading of the tortuosity score. The interpolation and filtering were optional steps. Segmentation was not needed in numeric phantoms without background noise. Details on the analysis steps are described below.

### Centerline cost functions

Cost functions for input into Dijsktra’s shortest path centerline algorithm included the modified distance from edge (MDFE) cost, center of mass (COM) and distance from edge (DFE)-COM. The DFE measures the distance of each segmented voxel to the nearest edge of the artery. The DFE exhibits a degeneracy which can interfere with centerline extraction: adjacent voxels may be equally distant from their nearest respective edges. The DFE is essentially a one-dimensional measure, ignoring all other edge locations but one in its calculation. The MDFE was developed [12] to use local spatial information to break ties between adjacent voxels with the same DFE values.

The COM function is computed by iteratively moving each voxel toward the current COM of its adjacent neighbor voxels, effectively collapsing the object inward. For the objects considered in this study, 30 iterations of motion toward the center of mass were sufficient. Each iteration uses the previous iteration’s mean positions and the cumulative distance moved by each voxel is recorded. To calculate the COM cost for each voxel, the cumulative distances moved were divided by the minimum non-zero distance moved in the entire segmentation and the result was cubed. Voxels at the segmentation edge moved farther, generating higher cost and voxels near the center moved shorter distances, generating lower costs. Because the COM calculation depends on the relationship between each voxel and its neighbors, it is highly sensitive to the shape of the object, eliminating most of the degeneracies encountered with the DFE algorithm.

The DFE-COM cost function combines the two cost functions. During the iterations of the COM algorithm, rather than assigning uniform weights to neighbor voxels, a weighted center of mass was computed using the DFE values as weights. Weighting gave more influence to the voxels with higher DFE in the middle of arteries when calculating the COM cost function.

### Numeric phantom generation

**Figure 1:**
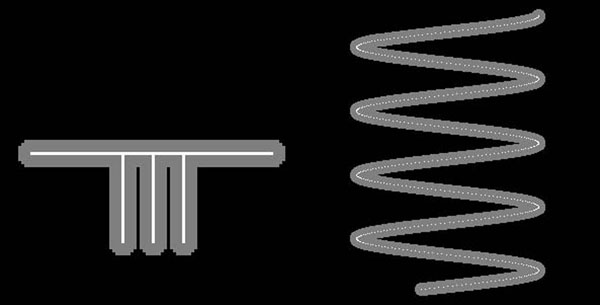
**Numeric phantom generation.** (left) The comb phantom was made from a comb shaped centerline. (right) A helix phantom was made from a helical centerline.

Numeric phantoms were generated by beginning with defined single point width centerlines. The centerlines were then discretized and placed within a discrete image volume. All voxels within a pre-defined radius of the centerline voxels were identified as object voxels, simulating imaged arteries. A subset of the discrete centerline locations were then used as positive controls for comparison with subsequent centerline extraction (Figure 1).

### Centerline stability and accuracy

The stability and accuracy of the DFE-COM cost-function centerline algorithm was measured and compared to the separate MDFE and COM cost function centerline algorithms on a set of numeric phantoms. The first numeric phantom considered was a comb phantom with a three voxel radius. A second series of branching phantoms with increasing image noise as designed by Aylward et al. [15] was also studied. Finally, the stability and ability of each algorithm to calculate centerlines around the internal carotid artery (ICA) siphon loop were tested with eight 3.0 T brain MRA image volumes.

### Tortuosity measurement

Tortuosity was determined at every point along the selected centerlines with the DFM [5,8] creating tortuosity curves. A single tortuosity measure was taken from each tortuosity curve either at the end of centerline or where the DFM was a maximum. Selection of the DFM value depended upon properties of the arteries being measured and is described in detail later.

### Tortuosity measurement of phantoms

**Figure 2:**
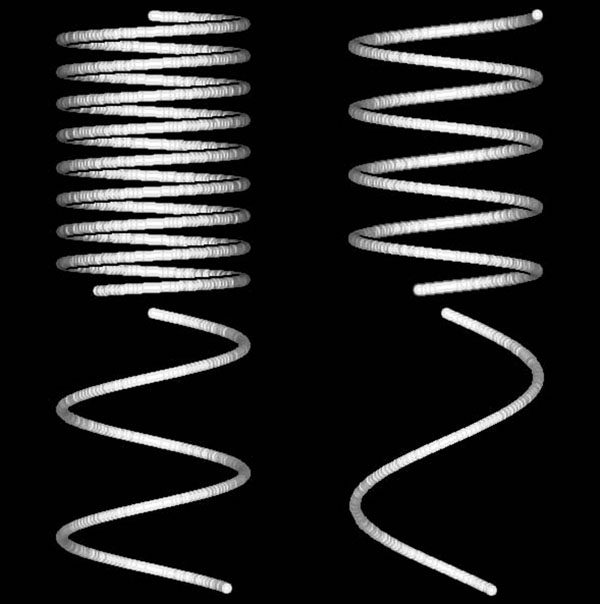
**Phantom helices.** 3-D helix phantoms were display with shaded surfaces. (top left) Pitch 5(2π). (top right) Pitch 10(2π). (bottom left) Pitch 20(2π). (bottom right) Pitch 40(2π).

The DFM tortuosity measurement was tested on 3-D numeric helix phantoms of increasing pitch with the DFE-COM centerline tortuosity measurement. The helix phantoms were generated by drawing a line with the equation h(*t*) = [r*cos(*t*), r*sin(*t*), (p*t)/(2π)] where *r* was the helix radius and *p* was the pitch of the helix and the radius of the simulated arterial width was 6 voxels. The helix radius *r* was fixed at 100 and four helices were generated with pitches 5(2π), 10(2π), 20(2π) and 40(2π) (Figure 2). The quantitative DFM tortuosity scores were taken at the highest peak of the tortuosity curves.

### Segmentation

**Figure 3:**
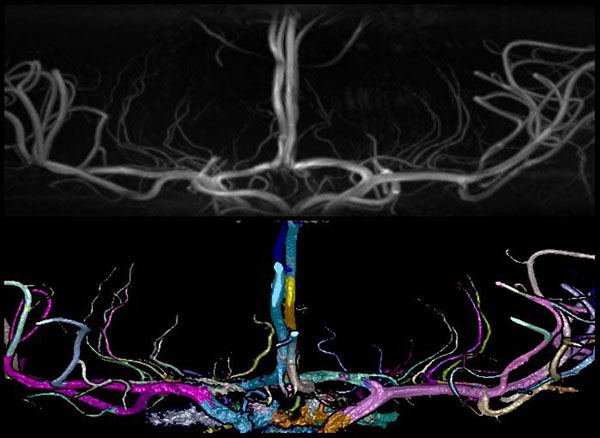
**High resolution artery segmentation.** (top) A high-resolution magnetic resonance angiography normotensive image was shown in maximum intensity projection. (bottom) Segmentation of the arteries was shown in shaded surface with colors to highlight bifurcations.

The arteries in the MRA images were segmented from background (Figure 3) using the Z-buffer segmentation (ZBS) algorithm [16]**.** ZBS algorithm works based on the assumption that arteries are the brightest structures in the image, they are sparsely represented in the image volume, and that bright artery voxels will be spatially close together. The algorithm casts rays in the Z axis through the 3D image volume finding the z-position of the brightest voxel in each ray. The z-positions of clusters of brightest voxels are then used as seeds for region growing and artery segmentation [16]. The artery segmentation is grown from the seed voxels by iteratively adding all neighboring voxels with intensities over a pre-determined intensity threshold. The intensity threshold was set as the 20^th^ percentile of all intensities of the seed voxels. Bubbles in the segmentation caused by low intensity slow moving or recirculating blood were filled using connected component analysis [17]. Small holes at the edges of the segmentation were filled by iterative reclassification. In three iterations, hole voxels were filled when they were surrounded by arterial voxels within 8 voxel steps along rays in 24 of 26 directions [12]. Finally connected component bubble filling was repeated.

### Human source images for hypertension tortuosity study

The hypertensive subjects were drawn from two populations. Twenty hypertensive subjects were identified by measurement of blood pressure at the Neuroscience Research Institute (NRI), Gachon University of Medicine and Science in Incheon, South Korea [3] and twenty negative controls were collected in the same study under approval from the Gachon University institutional review board and the Korean Food and Drug Administration.

For the second population, MRA images were selected from existing brain MRA images acquired from clinical hypertensive patients between 2008 and 2010 at the University of Utah Medical Center. The selection of subjects and the retrospective study of previously acquired images were performed with approval from the University of Utah institutional review board. All the Utah hypertensives (N =21) had a history of hypertension in the medical record demonstrating that they are under the care of a physician, making this a controlled hypertensive population. Diagnoses commonly associated with hypertension were allowed in the Utah hypertensive case population including transient ischemic attack, ischemic stroke, arterial disease, heart disease, sleep apnea and atrial fibrillation. Other diseases that may independently affect vasculature were excluded from the Utah hypertensive case population. These were diabetes, cancer [18], intracranial aneurysm, and genetic syndromes: hereditary hemorrhagic telangiectasia, Marfan syndrome and Loeys-Dietz syndrome [19,20]. The Utah negative control population was collected with IRB approval from clinical brain MRA images acquired from 2008 to 2010 (N = 45). The Utah negative control population had the following traits: subjects with headache, trigeminal neuralgia or head trauma; available brain MRA head images; no vascular pathology recorded in the radiology report; and no indication of the above listed diseases associated with hypertension in the subjects' medical records.

A third negative control population was obtained from a study on healthy aging conducted in North Carolina, U.S.A. [21]. Vascular and psychological diseases were screened out in this sample.

### MRI scanners

The data were acquired on different MRI scanners. The NRI data set was acquired with a 7.0 T MRI scanner (Magnetom, Siemens Medical Systems, Erlangen, Germany) [22,23]. The North Carolina data was acquired with a 3.0 T MRI scanner (Allegra, Siemens Medical Systems). The Utah images were clinical scans from both 1.5 T (GE) and 3.0 T (Siemens) MRI scanners at a range of image resolutions.

### Arteries measured

**Figure 4:**
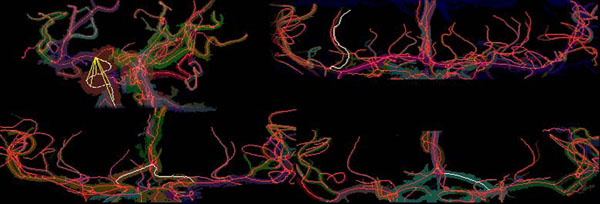
**Selected centerlines.** Centerlines of arteries and selections in white for tortuosity measurement were shown in maximum intensity projection (MIP). (top left) Right ICA was selected in white with progressive distance *d* in yellow. (top right) A lenticulostriate artery (LSA) of a normotensive subject was selected. (bottom left) The left to right ACA of a hypertensive patient was selected. (bottom right) The anterior cerebral artery (ACA) of a hypertensive subject was selected.

**Figure 5:**
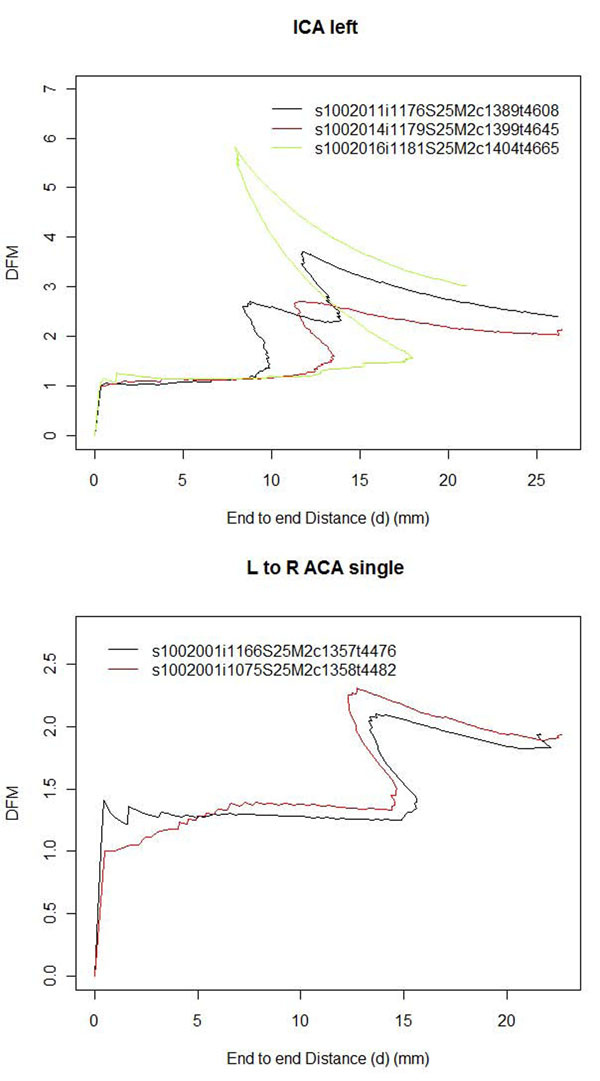
**Artery tortuosity curves.** Tortuosity curves of one subjects. (top). Tortuosity curves of three different subjects ICA arteries. The black curve is from Figure 3 top left. Tortuosity rises and falls. (bottom). Repeated tortuosity curves of the left to right ACA artery of the same subject, from figure 3 bottom left, measured from different MRA images.

The arteries measured, the start and end points of the centerlines considered, and the points along the tortuosity curve selected for tortuosity measurement are described in Table 1. Examples of artery selection are shown in Figure 4. The measurements for the lenticulostriate arteries (LSA) were for the left-most, right–most, and a mean of up to four prominent LSAs. Figure 5 demonstrates the tortuosity curves created for an internal carotid artery (ICA) with the DFM measurement taken from the peaks of the curves (Figure 5 top) and the left anterior cerebral artery (ACA) – anterior communicating artery (Acom)– right ACA (Figure 5 bottom) measurement taken from the ends of the curves.

**Table 1:** Tortuosity curve measurement point

Artery	Start	End	Measurement
Left ACA	Left ICA/ACA bifurcation	Acom	End to end
Right ACA	Right ICA/ACA bifurcation	Acom	End to end
Left ACA - Acom - Right ACA	Left ICA/ACA bifurcation	Right ICA/ACA bifurcation	End to end
Basilar	Posterior cerebrals	Vertebral arteries	End to end
Left ICA and Right ICA	ACA/MCA bifurcation	Bottom of slab	Peak
Left and Right VA	Basilar artery	Bottom of slab	Peak
LSA (7 T images)	MCA	Visible end	Peak and end-end

The measurement for each artery is taken from different points on the tortuosity curve of the anterior cerebral artery (ACA), internal carotid artery (ICA), anterior communicating (Acom), vertebral artery (VA), basilar arteries and lenticulostriate artery (LSA). Middle cerebral artery (MCA) bifurcations are used a starting points for some measurements.

### Filtering the images

The NRI data required filtering before segmentation. The image data was median filtered then the filtered image was subtracted from the original image. The arteries were segmented from the subtracted image. The effect of filtering on tortuosity was tested by measuring tortuosity of the Korean hypertensive population treated with different filters: no median filter, 5x5 median filter and an 11x11 median filter. After the comparison the 5x5 median filter was selected and used for treating the NRI data before segmentation. An exception was made where no filtering step used on segmentations used for measuring the small LSAs.

### Resolution and interpolation

The images were acquired at several different resolutions. Lower resolution images were sinc interpolated to higher resolutions [24]. For each subject in the NRI population, two MRA data sets were acquired: a thicker resolution (low 0.8x0.8x0.8 mm) set and a thinner and higher resolution (0.23x0.23x0.36 mm) set. The lower resolution data was interpolated to resolutions of 0.4x0.4x0.4 mm and 0.2x0.2x 0.2mm. The clinical Utah data was acquired from 0.38x0.38x1.6 mm (and interpolated on the scanner to 0.19x0.19x0.8) to 0.52x0.52x1.0 mm resolution and the lower resolution images were 2X interpolated to higher resolution (0.52x0.52x1.0 to 0.26x0.26x0.5 mm). The North Carolina data was acquired at 0.5x0.5x0.8 mm resolution and interpolated to 0.25x0.25x0.4 mm.

The effect of resolution and interpolation were measured in the NRI data set by measuring tortuosities of the same arteries for the same subjects at 0.4x0.4x0.4 mm, 0.2x0.2x0.2 mm and 0.23x0.23x0.36 mm resolutions. The 0.4x0.4x0.4 mm, 0.2x0.2x0.2 mm were interpolated from the same data acquisition and the 0.23x0.23x0.36 mm were acquired separately. The ICA arteries are only in the thicker transverse lower resolution volume limiting the testing of resolution effects to between the 4X (0.2x0.2x0.2 mm) and 2X (0.4x0.4x.4 mm) interpolations of the lower resolution image.

### Tortuosity comparisons

Arterial tortuosity was measured and compared between different data sets and sub sets. Sample data information was stored in a MySQL (http://www.mysql.com/) relational database coupled to the R statistical system [25] for visualization and statistical analysis. Comparisons were tested with the Wilcoxon rank-sum test. The tortuosity comparisons were between: different median filter subtractions of the NRI data, all resolutions of NRI data; the three negative controls; males and females; the NRI hypertensives and negatives; and Utah hypertensives and negatives.

## Results

### Centerline stability and accuracy

**Figure 6:**
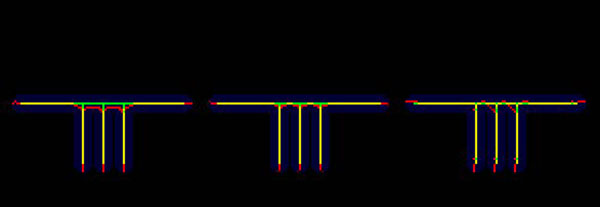
**Comb phantom.** Comb phantom where green is the true centerline, red is the algorithm calculated centerline and yellow is where the true and calculated centerline overlap. (left). The COM accuracy was displayed. (center) The MDFE accuracy was displayed. (right) The DFE-COM accuracy was displayed.

Centerline accuracy was measured by comparing the measured centerline with the true centerline in the numeric phantoms and stability was measured by testing the centerlines found with different starting points. The results for the numeric phantoms are summarized in Figure 6 and Tables 2 and 3. The multiple branches of the comb phantom (Figure 1 left) pulled the COM centerline in red below the true centerline in green (Figure 6 left). The MDFE cost (Figure 6 middle) and DFE-COM (Figure 6 right) centerlines overlap (in yellow) more with the true green centerline than the COM centerline.

**Table 2:** Comparison of algorithm stability and accuracy of comb phantom

Algorithm	Number of trees	Stability	RMSE of Accuracy
COM	6	0.918	0.879
MDFE	6	0.819	0.417
DFE-COM	6	0.905	0.413

The COM cost function shortest paths centerline algorithm was less accurate in the comb phantom than the MDFE cost and DFE-COM cost function algorithms.

**Table 3:** Comparison of algorithm Stability and accuracy on 3 branch phantom

Phantom noise	Algorithm	Number of trees	Stability	RMSE of Accuracy
SD-10	COM	3	0.960	0.463
SD-10	MDFE	3	0.930	0.393
SD-10	DFE-COM	3	1.00	0.556
SD-20	COM	3	0.950	0.528
SD-20	MDFE	3	0.946	0.674
SD-20	DFE-COM	3	0.955	0.561

The DFE-COM cost function shortest paths centerline algorithm had similar stability and accuracy to the COM based centerline algorithm. The MDFE cost algorithm accuracy degraded going from standard deviation (SD) 10 to 20 noise.

Centerline stability as a function of cost function algorithm on brain MRA images is summarized in Table 4. The DFE-COM was tested on the ICA siphon loop, visualized with a white centerline in Figure 4 top left, where it loops back upon itself often kissing itself and causing problems for centerline extraction. The DFE-COM was able to complete as many ICA siphon loops as the COM algorithm with similar stability.

**Table 4:** Comparison of centerline algorithms on MRA brain images

Algorithm	ICA siphons accurate	Portion ICA siphons correct	Both ICA correct in image	Portion correct images	Mean number of trees	Standard deviation of trees	Mean stability	Standard deviation stability
COM	15/16	0.938	7/8	0.875	37.000	12.352	0.872	0.0459
MDFE	7/16	0.438	1/8	0.125	39.875	13.228	0.673	0.0732
DFE-COM	15/16	0.938	7/8	0.875	38.625	11.439	0.825	0.0434

The COM and DFE-COM cost function shortest paths centerline algorithms calculated the correct centerline in the same number of correct ICA siphons.

### Tortuosity measurement of phantoms

**Figure 7:**
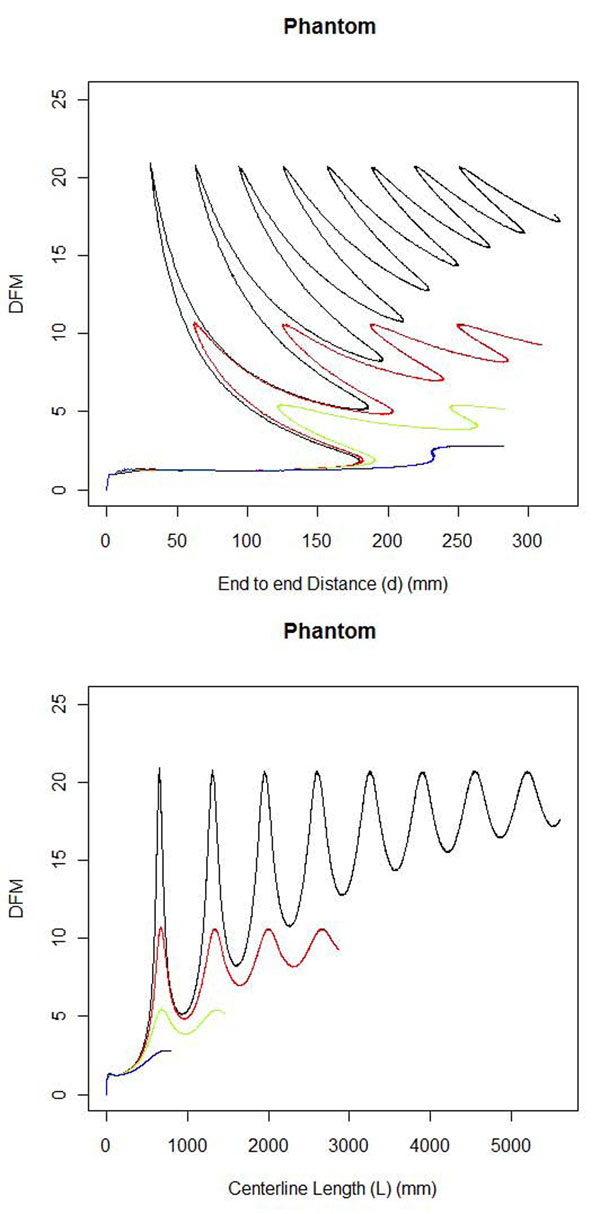
**Phantom tortuosity curves.** Helix phantom tortuosity curves are shown in maximum intensity projection. The pitch 5(2π) (black), pitch 10(2π) (red), pitch 20(2π) (green) and pitch 40(2π) (blue) phantoms decrease in tortuosity. (top) DFM tortuosity plotted versus distance *d* from the start. (bottom) DFM tortuosity plotted versus Length *L* from the start.

The DFE-COM centerline DFM tortuosity scores were higher for tighter coiled helix phantoms with lower pitches and the tortuosity scores increased proportionally to the increase in the number of coils (Table 5). The pitch 40(2π)-helix has approximately one coil (DFM = 2.48) and the pitch 20(2π)-helix has approximately two coils and has almost double (ratio = 1.95) the tortuosity score (DFM = 5.45). The tortuosity curves were displayed with the distance *d* (Figure 7 top) or length *L* (Figure 7 bottom) on the x-axis showing the rise and fall of the tortuosity curve.

**Table 5:** Helix Phantom tortuosity

Phantom	Peak Distance Factor Metric	Approximate coils	Peak DFM/2.80 ratio
Pitch 5(2π)	20.95	8	7.48
Pitch 10(2π)	10.76	4	3.84
Pitch 20(2π)	5.45	2	1.95
Pitch 40(2π)	2.80	1	1.00

The peak distance factor metric tortuosity scores are lower with increasing pitch of the three-dimensional helix phantoms.

### Median filter effect

**Figure 8:**
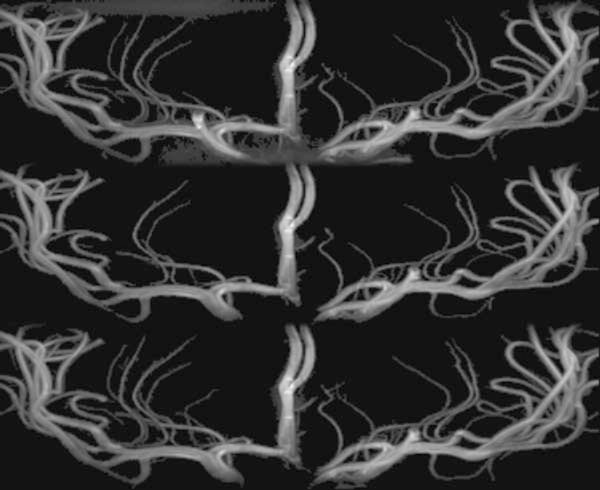
**Median filtered segmentations.** Median filter subtraction segmentations of a 7.0 T NRI image of a hypertensive patient were displayed in maximum intensity projection. (top) Segmentation without the median filter subtraction left background noise in the segmentation. (center) Segmentation with the 5x5 median filter subtraction removed background noise and smaller lenticulostriate arteries (LSA). (bottom) Segmentation with the 11x11 median-filter leaves small amounts of background noise near the larger arteries while leaving the LSAs in the segmentation.

The 7.0 T images were segmented with no suppression of background noise under the ZBS segmentation algorithm shown in Figure 8, top. Subtracting a median filtered image from the images as the first step in the segmentation removed the background noise from the segmentation but also removed some of the small lenticulostriate arteries while leaving the larger arteries especially in the case of the 5x5 median filter (Figure 8 middle). The larger 11x11 median filter removed most background noise but left some noise near the larger arteries while leaving most LSAs in the segmentation (Figure 8 bottom).

The median filter subtractions (none, 5x5 and 11x11) had no significant effect on tortuosity measurements of left ACA, right ACA, left to right ACA, left ACA and right ACA arteries of the hypertensive Korean population at the β = α/n = 0.05/8 = 0.00625 significance level with a two-sided Wilcoxon rank-sum test.

### Resolution and interpolation effect on tortuosity

**Figure 9:**
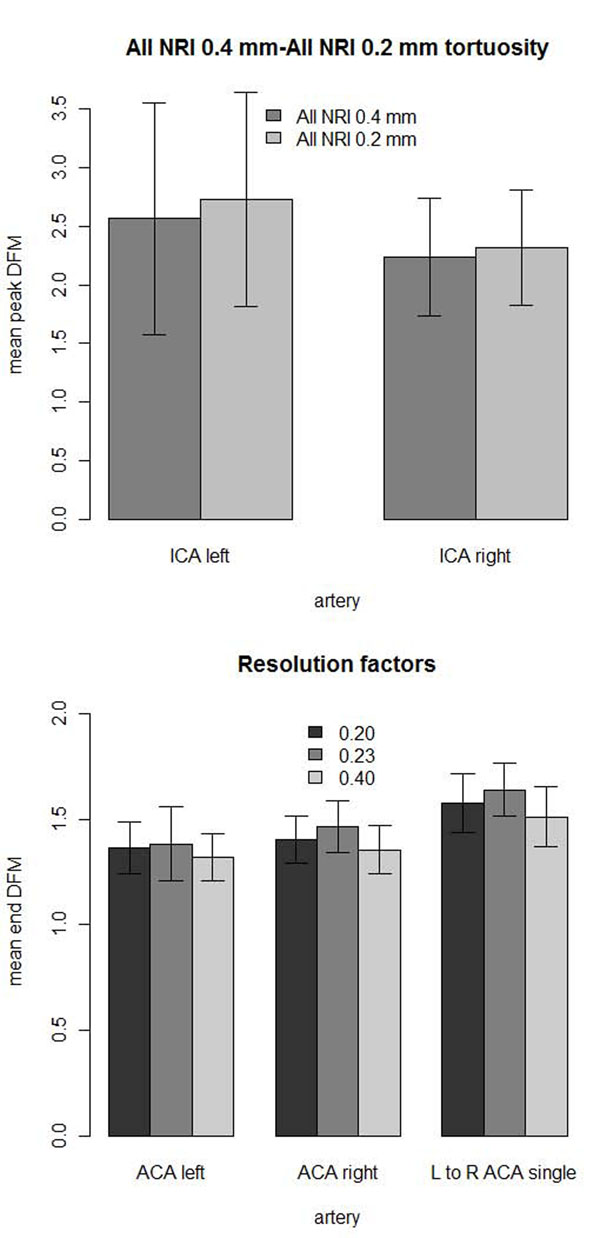
**Tortuosity of resolutions.** Comparison of mean tortuosity of the same Korean subjects from NRI with one standard deviation error bars. (top) Left and right ICA tortuosity measurements from higher 0.2x0.2x0.2 mm (0.2 mm) resolutions interpolations of the same images increased compared to 0.4x0.4x0.4 mm (0.4 mm) resolution. (bottom) The mean DFM tortuosity of the 0.2x0.2x0.2 mm (0.2) and 0.23x0.23x0.36 mm (0.23) resolution images were closer together than to the 0.4x0.4x0.4 mm images (0.4).

The tortuosity was measured for the Korean hypertensive and negative control populations from the low and high-resolution images. The image volumes were not all long enough to capture the ICA accounting for low numbers of ICA measurements. Out of the total population size of 40 there were: 19 2X interpolated left ICA, 19 2X interpolated right ICA, 21 4X interpolated left ICA and 23 4X interpolated right ICA. The tortuosity values were compared with a 2-sided Wilcoxon rank-sum test, and a paired 2-sided Wilcoxon rank-sum test on all cases where measurements were made on both the high and low interpolations of the same artery. The 4X interpolation had 6.40% higher left ICA (P = 0.294, paired P = 0.00042) and 3.65% higher right ICA (P = 0.452, paired P = 0.0348) tortuosity than the 2X interpolation (Figure 9 top). The mean resolution of 0.23x0.23x0.36 mm (mean 0.273 mm) is closer to 0.2x0.2x0.2 mm (0.0733 mm difference) than 0.4x0.4x0.4 mm (0.127 mm difference). The mean DFM taken from the tortuosity curves of the left ACA, right ACA and left to right ACA of the 0.23x0.23x0.36 mm images was 6.89±2.45% greater than the 0.4x0.4x0.4 mm images of the same subjects. The 0.2x0.2x0.2 mm images actually had 3.05±1.89% lower tortuosity than the 0.23x0.23x0.36 mm images. The difference in magnitude of both mean resolution and tortuosity between 0.2x0.2x0.2 mm and 0.23x0.23x0.36 mm images was smaller than between the 0.4x0.4x0.4 mm and 0.23x0.23x0.36 mm images (Figure 9 bottom). Due to increased similarity of scores, only the 0.2x0.2x0.2 mm and 0.23x0.23x0.36 mm were used for the hypertensive and negative control comparison experiments later in this study.

### Comparison of negative control populations

**Figure 10:**
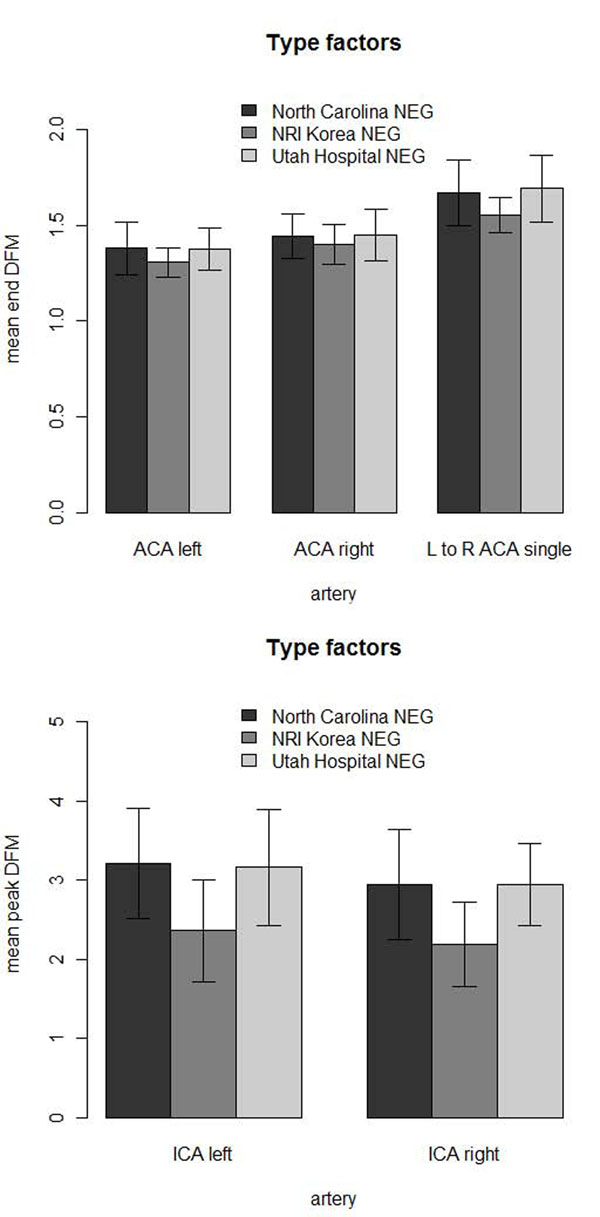
**Negative control tortuosities.** Utah and North Carolina negative (NEG) controls had significantly higher tortuosity than the NRI Korean negative controls: (top) mean end tortuosity measurements, (bottom) mean peak tortuosity measurements.

The Korean negative control population showed significantly less arterial tortuosity compared to arteries of the Utah and the North Carolina negative controls (Figure 10) and the three populations were of similar age (Table 6). The Utah and North Carolina negative controls did not have significantly different arterial tortuosity. ANOVA analysis of the three negative controls: NRI Korean, North Carolina and Utah hospital showed significant differences in the left ACA, left to right ACA, left ICA, and right ICA arteries at the β = α/*n* = 0.05/5 = 0.01 level. Pair-wise comparisons between the negative controls with a 2-sided Wilcoxon rank-sum test showed the Korean population had significantly lower tortuosity of the left to right ACA, left ICA and right ICA than North Carolina and Utah hospital population at the β = 0.01 level. The North Carolina and Utah populations did not show any significant differences in arterial tortuosity.

**Table 6:** Negative control demographics

(-) control	Total	Mean age	Male (%)	Female (%)	White	Asian	Black
NRI Korean	20	47.7	3(15.0)	17 (85.0)	0 (0.00)	20 (100.0)	0 (0.00)
North Carolina	95	42.7	45 (47.4)	50 (52.6)	83 (87.4)	8 (8.4)	4 (4.2)
Utah	45	46.7	23 (51.1)	22 (48.9)	-	-	-

The Korean negative control population was mostly female whereas the North-Carolina and Utah negative control populations where evenly split between females and males.

### Female and male comparisons

The Utah and North Carolina negative populations were split evenly between males and females while the Korean negative population was mostly female. Ethnicity was rarely indicated in the Utah medical record but based on the composition of the state of Utah, the subjects are most likely white European descent. The North Carolina population was mostly of white European descent (Table 6). The differences between the Korean control and the other two control populations were Asian ethnicity versus white European descent and a greater percentage of females.

Male and female North Carolina and Utah populations showed no significant differences at the β = α/*n* = 0.05/5 = 0.01 level of 2-sided Wilcoxon rank-sum tests for five arteries measured. The lowest P-Value was of the left ICA (P=0.0288) of the North Carolina population where tortuosity values for males were higher than females and females had higher tortuosity in three of five arteries measured. There was no significant difference in tortuosity between males and females in eight arteries compared at the β = α/*n* = 0.05/8 = 0.0625 level in the Utah negative control (lowest P = 0.0114 with a 1.40% increase in male right VA tortuosity).

**Figure 11:**
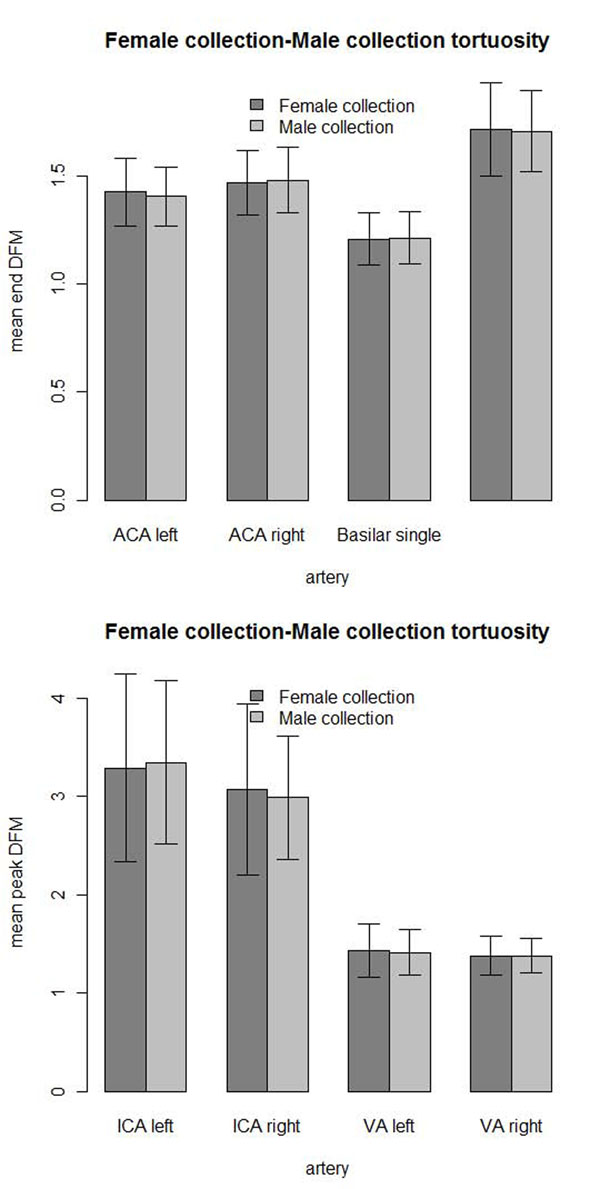
**Female-male tortuosity.** Mean arterial tortuosity comparison with 1 standard deviation error bars between female and male subjects showed no significant differences: (top) mean end DFM and (bottom) mean peak DFM tortuosity measurements.

There was no significant difference in arterial tortuosity between males and females in our entire current collection of tortuosity measurements at the α = 0.05 level (lowest P = 0.342) (Figure 11). The collection included the three negative controls, and subjects with vascular diseases. The diseases included hypertension, diabetes, cancer, stroke, intracranial aneurysm, hereditary hemorrhagic telangiectasia, Marfan syndrome and Loeys-Dietz syndrome subjects. There are more females (257) in the collection than males (185). The mean ages were similar for females (48.2) and males (46.5).

### Korean hypertension tortuosity comparison

**Figure 12:**
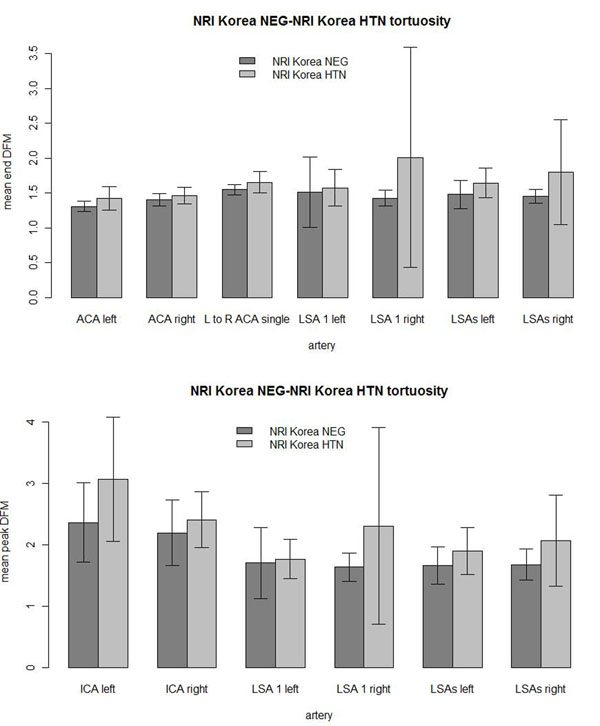
**NRI Korean tortuosities.** NRI Korean negative versus hypertensive (HTN) arterial mean tortuosity comparisons with 1 standard deviation error bars. LSA 1 left was the left most LSA and LSA 1 right was the right most LSA. (top) The figure showed the mean end DFM tortuosity measures. (bottom) The figure showed the mean peak DFM tortuosity measures.

The Korean hypertensive population had higher tortuosity across all 13 artery measurements than the Korean negative control (Figure 12). 10 were significant at the α = 0.05 level of the 1-sided Wilcoxon rank sum test. Even with the statistical correction of β = α/*n* = 0.05/13 = 0.0038, 5 of the 13 tortuosity measurements were significantly higher in the Korean hypertensive population. The most significant measurements were the left ACA (P= 0.00377), the end DFM of left LSAs (P = 0.000161), the end DFM of the right LSAs (P = 0.00052), the peak DFM of the left LSAs (P = 0.00977) and the peak DFM of the right LSAs (P = 0.00080). There were more prominent LSAs per subject in the negative control (3.50 left, 3.35 right) than in the hypertensive subjects (2.15 left, 2.30 right).

### Utah hypertension

**Figure 13:**
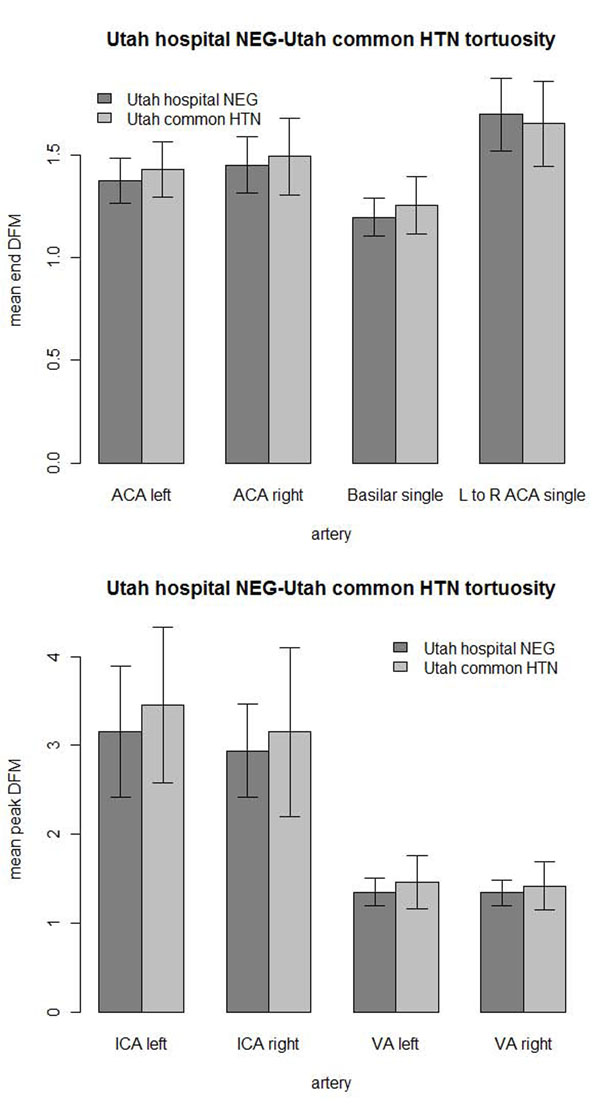
**Utah hypertensive tortuosity.** Comparison of Utah common hypertension (HTN) and Utah hospital negative (NEG) control with 1 standard deviation error bars: (top) mean end DFM tortuosity measures and (bottom) mean peak DFM measures.

The Utah hypertensive population (N=21) did not show significant increases in tortuosity compared to the Utah hospital negative control (N=45) at the β = α/*n* = 0.05/8 = 0.00625 level (Figure 13). The test was conducted only against the Utah negative control population. Not all images contained measurable arteries for all arteries examined. The number of measurements and statistical test results are in Table 7. An F-test of variances showed higher variance of the hypertensive Utah population tortuosity than the negative control of the right ICA (P = 0.00206), left VA (P = 0.00093) and right VA (P = 0.00174) at the β = 0.00625 level. The hypertensives were insignificantly higher in tortuosity of seven of the eight arteries compared.

**Table 7:** Utah retrospective tortuosity comparison

Artery	Negative (N)	Hypertensive (N)	1-sided Wilcoxon	2-sided F Test
Left ACA	43	21	0.0565	+0.232
Right ACA	39	21	0.279	-0.0824
Basilar	42	18	0.0641	+0.0302
L to R ACA	24	11	0.805	+0.501
Left ICA	35	19	0.132	+0.371
Right ICA	36	19	0.366	**+0.00206**
Left VA	36	18	0.283	**+0.00093**
Right VA	35	16	0.297	+0.00174

A (+) indicates increased hypertensive tortuosity and a (-) indicates decreased hypertensive tortuosity

## Discussion

We were able to develop a process of measuring arterial tortuosity including segmentation, filtering, interpolation, centerline extraction and DFM tortuosity analysis. The DFE-COM centerline was selected for making tortuosity measurements because it was able to calculate centerlines around most of the ICA siphon loops in a brain MRA data set and had better accuracy in the comb phantom. The subtraction of median filtered images from the MRA data had no significant effect on tortuosity and was used when necessary to improve artery segmentation. The 5x5 median filter was selected when measuring tortuosity of arteries other than the LSAs in the NRI data for the filter's ability to remove more fully background noise and process images faster than the larger filter while producing no significant change in tortuosity measurement. There was too much chance of data loss when measuring tortuosity of the LSAs to justify the use of the median filter. The DFM tortuosity curve consistently measured the increasing tortuosity of the helix phantoms. Interpolating lower resolution images to higher resolution reduced the effects of resolution on tortuosity measurement. These results led us to use interpolation when comparing tortuosity in hypertensive populations versus negative controls.

Our methods measured a statistically significant increase in arterial tortuosity in the NRI Korean hypertension population compared to the Korean negative control. We also observed a dependence of tortuosity measurements upon image resolution. Higher resolution images increased the DFM tortuosity scores. Interpolating lower resolution images to higher resolutions reduced or eliminated the reduction in tortuosity for lower resolutions. Finally different populations may have different baseline tortuosities.

Hypertension correlated with increased arterial tortuosity in the Korean population study. The consistency across the arteries measured suggests that increased arterial tortuosity with hypertension is a global phenomenon. Greater change in tortuosity was seen in the ICA and LSA arteries than in the ACA measurements. The ICAs are longer than the ACAs possibly allowing more twisting due to increased hypertension. The LSAs are narrower than the ACAs and small narrow arterioles have been shown to twist strongly in response to hypertension [1]. The LSAs also had the higher significance of the tortuosity increase. To simplify measuring arterial tortuosity for clinical use, measuring one longer or narrower diameter artery may suffice for gauging tortuosity.

The correlation between tortuosity and hypertension was not repeated in the Utah populations. The Utah hypertensive group was under physician care, therefore many patients may have been on anti-hypertensive medications making this a largely controlled hypertensive population. Lack of completeness in the medical records made the number of subjects on hypertensive medication difficult to determine. A future experiment could compare controlled versus uncontrolled hypertensive (when identified) populations to study if anti-hypertensive medications have an effect on arterial tortuosity. Another possibility is that the Utah Hospital negative control is not truly negative. However, the Utah Hospital negative control was similar to the North Carolina negative control population indicating that the Utah hospital control was negative for increased arterial tortuosity and that patients imaged for reasons other than vascular disease reasons are usable as negative controls.

In a retrospective analysis of images such as this one, universal negative controls may not be possible. The Korean population showed significantly lower tortuosity than the Utah population. The North Carolina negative control was similar to the Utah hospital population in tortuosity. The Korean negative control population was mostly female. Females in the Utah hospital and North Carolina negative controls and in the entire tortuosity collection did not show significantly lower tortuosity than the corresponding male populations. The Korean data was higher resolution than the North Carolina or Utah data after final interpolations. Any remaining resolution effect on tortuosity would increase the Korean data more than the others but they still had the lowest tortuosity of the negative controls. The Utah hospital and North Carolina populations were mostly white Americans of European descent and the Korean population was all Korean descent. Ethnicity remains as one possible cause of the decrease in Korean population tortuosity but the negative Korean control tested here was not a broad representation of the Korean population. With the ability to interpolate images taken at different resolutions we will attempt to obtain more ethnic populations from clinical images to compare arterial tortuosity to determine if ethnicity affects arterial tortuosity.

## Conclusions

The methods in the study were able to measure a correlation between hypertension and arterial tortuosity. The DFE-COM centerline algorithm was able to make centerlines for the arteries of interest. The median filter subtraction allowed segmentation of the high-resolution data sets without affecting tortuosity. A significant increase in arterial tortuosity was measured in the uncontrolled NRI Korean hypertensive population versus a corresponding negative control. The Korean hypertensive population was not representative of all hypertensive populations or even of all Koreans. No significant arterial tortuosity increase was seen in the controlled Utah hypertensive population. Therefore we do not know if the increase in tortuosity with hypertension occurs in all populations. These methods can be used to study more populations to find out more about the relationships between hypertension and arterial tortuosity.

## List of abbreviations used

ACA: anterior cerebral artery; Acom: anterior communicating artery; COM: center of mass; *d:* distance; DFE: distance from edge; DFM: distance factor metric; FOV: field of view; HTN: hypertensive; ICA: internal carotid artery; *L: left; L:* length; LSA: lenticulostriate artery; MDFE: modified distance from edge; MIP: maximum intensity projection; MRA: magnetic resonance angiography; MRI: magnetic resonance imaging; NEG: negative; R: right; T: Tesla; TOF: time of flight; ZBS: Z buffer segmentation.

## Competing interests

There are no competing interests.

## Authors' contributions

KTD developed the software, conducted the measurements and wrote the paper. JAR, DLP and RHS aided in designing the experiments and writing the paper. CKK and ZHC collected and provided the NRI data and contributed to the analysis of the results of the NRI data. All authors read and approved the final manuscript.
